# Primary Erythromelalgia Treated With 10% Capsaicin Cream: A Case Report and a 10-Year Follow-Up

**DOI:** 10.7759/cureus.28342

**Published:** 2022-08-24

**Authors:** James A Tolley, Laurence E Walsh

**Affiliations:** 1 Anesthesiology/Chronic Pain, Indiana University Health, Indianapolis, USA; 2 Anesthesiology/Chronic Pain, Indiana University School of Medicine, Indianapolis, USA; 3 Neurology/Pediatric Neurology, Indiana University Health, Indianapolis, USA; 4 Neurology/Pediatric Neurology, Indiana University School of Medicine, Indianapolis, USA

**Keywords:** pediatric pain, pediatric pain management, pain, axillary catheter, lumbar epidural, topical capsaicin, capsaicin, primary erythromelalgia, erythromelalgia

## Abstract

In this case report, we describe the difficulty in finding a suitable treatment for a nine-year-old girl with erythromelalgia. Initially, she could only find pain relief through immersion of her hands and feet in buckets of cool water. Her pain did not respond to outpatient treatments, and she was ultimately admitted to the hospital for pain management. Many different medications and modalities were tried over the course of several weeks in the hospital. Finally, she received the most benefit from 10% compounded capsaicin cream administered under general anesthesia with regional analgesia for post-application pain. Over the course of several years, exacerbations of her pain were treated with additional applications of 10% capsaicin cream, with each application providing relief for an increased duration. Her severe pain flares eventually went into remission after several years. Today, after more than a decade following her initial presentation, she is a successful college student and is taking no medications for her erythromelalgia.

## Introduction

Mitchell originally described a rare, painful affliction of the feet in 1872, which received little attention. Six years later, in 1878, he published an additional case series, mostly of male patients suffering from a red, painful hand and foot disorder that he conveniently labeled “erythromelalgia”, which he derived from the Greek for red (*erythros*), member (*melos*), and pain (*algos*). The disorder was invariably exacerbated by activity and warm weather, relieved by colder weather or immersion in cold water, and difficult to treat with the therapies of the day. Mitchell observed that the onset of the disorder was preceded by prolonged activity or disease, could vary in severity, and undergo periods of remission [1].

In 1938, Smith and Allen divided the disorder into primary, secondary, and idiopathic causes and proposed a new term “erythermalgia” to emphasize the importance of heat in the causation and symptomatology [2]. Since that time it has been determined that primary erythromelalgia may be associated with mutations in the SCN9A gene encoding for the Nav1.7 sodium channel [3] or other genetic associations [4]. Secondary erythromelalgia is associated with a variety of underlying conditions, including various myeloproliferative, connective tissue, vascular, neurologic, and other disorders, as well as exposures to certain drugs [5].

One of the hallmarks of erythromelalgia is that it is refractory to treatment [6], and many different medications and therapies have been used with varying degrees of success [7, 8]. The following case report illustrates the difficulty in finding a satisfactory treatment regimen and describes the rare use of compounded 10% topical capsaicin in the treatment at the initial presentation and several subsequent flares in a pediatric patient with primary erythromelalgia.

## Case presentation

We present the case of a nine-year-old Caucasian female who was admitted to a tertiary pediatric hospital complaining of a three-week history of bilateral hand and foot pain that began in the toes as a burning and itching sensation. Prior to admission, she had been seen by her primary care physician and local emergency department and had trialed acetaminophen with codeine, ibuprofen, diazepam and hydroxyzine, none of which had helped with her pain. She denied any redness or swelling of the hands or feet at the time. She had noticed a great deal of pain relief upon immersion in cool water.

Neurology was consulted and suggested mexiletine on day two of admission for a presumed diagnosis of erythromelalgia based upon clinical criteria following unremarkable nerve conduction studies of both upper and lower extremities. Nerve conduction studies of the left arm revealed normal median and ulnar sensory responses. In the left leg, the peroneal conduction velocity was mildly slow (42.0 m/s) and sural response was low amplitude with prolonged distal latencies (7.3 ms for peroneal and 5.9 ms for sural). It was noted, however, that the patient’s hands and feet were extremely cold due to the cold-water immersion and she did not tolerate warming. She was also started on morphine, which helped with sleep due to its sedative effects. 

On day three, methylprednisolone was begun but this was stopped 24 hours later due to psychosis and hallucinations. On day six, mexiletine was replaced by a lidocaine infusion. Aspirin was added and a peripherally-inserted central catheter (PICC) was placed for ongoing lidocaine infusion. On day nine, gabapentin was started at bedtime. On day 11 of admission, she was taking aspirin, lorazepam, gabapentin, and hydrocodone with acetaminophen in addition to the lidocaine infusion. Despite these measures, she still required nearly continuous immersion of her hands and feet in basins of cold water for relief.

On day 12, hydrocodone was replaced with sustained-release morphine and immediate release morphine for breakthrough pain. Oral clonidine and compounded ketamine and amitriptyline cream were also added. She remained on a lidocaine infusion. For the first time in four weeks, she was able to sleep nine hours through the night and spent less time with her hands and feet in water.

On day 15, sertraline was started and the lidocaine infusion rate was decreased. She was able to engage in art therapy, but on day 16 she again had pain in her elbows and forearms so the lidocaine infusion rate was increased. Discussions between neurology and the pain team led to placement of bilateral axillary catheters and a lumbar epidural catheter under general anesthesia on admission day 18. Lidocaine and clonidine were infused through the catheters, and she was able to stop using cool water for pain. She was also receiving application of capsaicin 0.025% cream four times daily. A dexmedetomidine infusion that was started 48 hours earlier was stopped on day 19. 

On day 20, 8% capsaicin patches were applied to both hands and feet under conscious sedation in the patient’s room. On day 21, the right axillary catheter fell out, and the next day, morphine and ketamine/amitriptyline creams were restarted. Pain in the hands was reduced to 1/10 but pain in the feet remained elevated, and the patient refused to ambulate.

By day 26 of admission, the patient’s hand pain was well controlled, and she had not used water on her hands or feet in three days. She was able to wear shoes and do some walking, but this remained limited by her foot pain. After discussions between the anesthesia pain service and neurology, it was decided that 10% capsaicin cream application would be performed under general anesthesia with a caudal block performed to provide post-application analgesia on day 29. The cream was compounded by the pharmacy and applied to both feet up to the ankles. Her feet were wrapped in Kerlix gauze for 45 minutes and then washed multiple times with Ivory soap and water. During the ensuing 24 hours, she did not use any prn medications for pain.

Over the next two days, she continued to do well but still had problems with fine motor control such as buttoning a shirt. The patient was discharged on day 32 on a regimen of four days of sustained-release morphine to complete a wean, sertraline 50 mg daily, acetylsalicylic acid (ASA) 81 mg three times a day (TID), and gabapentin liquid 250 mg TID (10 mg/kg). She also was given prescriptions for prn morphine, diphenhydramine, lorazepam and hydrocortisone cream. Physical and occupational therapy were undertaken as an outpatient.

Decade of follow-up

At her initial three-month follow-up appointments with the chronic pain service and neurology, she was doing well. She reported short bursts of a shocking sensation each hour which did not limit activity. She continued to have significant distal atrophy and mild contractures of the hands. She was maintained on sertraline and pregabalin and was participating in physical therapy (PT) and occupational therapy (OT) weekly. Her level of activity continued to increase through her follow-up appointment at five months post-discharge.

Over the next few weeks, she began having increased foot pain followed by increasing pain in the hands and decreased activity. She stopped walking and attending school. By month six, she returned to the operating room for application of 10% capsaicin cream under general anesthesia with bilateral axillary blocks and a caudal block for post-application analgesia. Cream application was as before and was placed on both feet and hands. She was observed overnight and did well. By the next morning, her pain and shocking sensations were gone and she was sent home.

At approximately six months after presentation, her nerve conduction studies were repeated. This again showed normal median and ulnar sensory responses but absent median, peroneal, and tibial motor responses. Ulnar motor response had mildly low amplitude but normal conduction velocity and latency. Sural response was absent. This was now interpreted as abnormal and consistent with a length-dependent peripheral neuropathy with motor predominance. This had progressed from her initial nerve conduction study.

In month 19 after her initial admission, she began having increasing hand and foot pain such that she presented for a repeat treatment consisting of another 45 minute application of 10% capsaicin cream under general anesthesia with bilateral axillary blocks and a caudal block for post-application analgesia. She was discharged on sertraline 50 mg daily and pregabalin 50 mg twice a day (BID).

In month 35, she had 10 days of increasing hand and foot pain so she underwent a third 45 minute application of 10% capsaicin cream to both hands and feet under general anesthesia with bilateral supraclavicular nerve blocks and a caudal block for post-application analgesia.

A follow-up during month 44 revealed that she was pain free and experienced no shocking sensations. Two months later, after a week of increased hand and foot pain requiring cold water immersion, she presented for her fourth and final capsaicin treatment of the hands and feet which was identical to her other outpatient treatments. The next month, she was engaged in PT and OT, pain free, and not taking any medications. She did have reducible contractures of the hands and wasting of the intrinsic hand muscles with 4- to 4/5 grip strength. Her gait was normal.

Now, over a decade after her initial presentation and more than seven years after her most recent capsaicin treatment, she remains on no medications. At baseline, she will notice “shocks” a few times per day which can increase to up to 20 times per hour with illness. At times, these shocks will serve as a prodrome to an upper respiratory infection. During times of severe physical or emotional stress, mini flares of pain, redness, and heat can last for several hours. She currently has no physical limitations and is an active college student.

## Discussion

In 1932, Brown listed six criteria required for the diagnosis of erythromelalgia [6,9]: localized redness and burning pain; bilateral involvement; produced and exacerbated by exercise and heat; relief of symptoms by rest, elevation, and cold; absence of a concomitant condition to explain symptoms; refractory to treatment. Our patient met these criteria. In terms of symptomatic treatment of pruritus and severe pain, the patient was trialed on over 20 different medications with various mechanisms of action prior to successful management with 10% compounded capsaicin cream (Table 1). These drugs were administered topically, orally, intravenously, or utilizing regional analgesic techniques.

**Table 1:** Medications trialed in our patient for erythromelalgia ASA - acetylsalicylic acid, NSAIDs - non-steroidal anti-inflammatory drugs, NMDA - N-methyl-D-aspartate, TRPV1 - transient receptor potential cation channel subfamily V member 1

Drug class	Individual drugs used
NSAIDs	acetaminophen, ASA, ibuprofen
Local anesthetics	mexilitine, lidocaine, ropivacaine
Alpha-2 agonists	clonidine, dexmedetomidine
Opioids	codeine, morphine, hydrocodone, fentanyl, hydromorphone
Anticonvulsants	gabapentin, pregabalin
Antidepressants	amitriptyline, sertraline
Benzodiazepines	diazepam, lorazepam
Antihistamines	hydroxyzine, diphenhydramine
Steroids	methylprednisolone, hydrocortisone
NMDA antagonist	ketamine
TRPV1 agonists	capsaicin

A survey of 41 members of the Erythromelalgia Association published by Cohen in 2000 lists 19 different medications or classes that had been tried in addition to topical medications, a morphine pump, spinal cord stimulator, and non-pharmacologic treatments such as biofeedback and acupuncture [7]. Many of the medications were not helpful, although gabapentin was reported to be beneficial in all 16 of the members who tried it. A separate retrospective review of 32 pediatric cases at the Mayo Clinic over almost four decades [8] similarly lists multiple medications and treatments demonstrating a lack of efficacy for any particular treatment modality. Of note is that gabapentin was not helpful in four of the six patients in which it was tried. Likewise, our patient’s pain was quite resistant to treatment.

As a result of her family’s wishes, the patient has not undergone molecular testing. Given her age at presentation and lack of identifiable underlying systemic disorders, we believe that it is very possible that she harbors a pathogenic variant in the SCN9A gene. In a recent systematic review of pediatric erythromelalgia and SCN9A mutations, Arthur et al. [3] found that 12 of 25 patients with SCN9A-associated erythromelalgia had a family history of erythromelalgia, although those with a negative family history had inconsistently reported confirmatory genetic testing of family members. This suggests that up to half of the cases of primary erythromelalgia may arise de novo (i.e., via germline mosaicism in an unaffected parent, somatic mosaicism in a mildly affected parent, or post-zygotic mutation in the affected child). Conversely, even in inherited cases of erythromelalgia, many patients can be negative for a pathogenic mutation of SCN9A [10], suggesting genetic heterogeneity for the disorder.

The family of SCN genes encodes nine voltage-gate sodium channels that are present in the peripheral and central nervous systems, heart, and muscle. Mutations in these genes have been associated with pain disorders, epilepsy, developmental disabilities, familial hemiplegic migraine, episodic ataxia, cardiac conduction disorders, and periodic paralyses [3,11]. The SCN9A gene encodes the sodium channel Nav1.7, which is primarily located in the peripheral nociceptive neurons whose cell bodies are located in the dorsal root ganglion as well as the trigeminal and sympathetic ganglions and is important in the transmission and processing of pain information [3]. Phenotypes associated with gain-of-function mutations in SCN9A include primary erythromelalgia, generalized epilepsy with febrile seizures plus (GEFS+), familial febrile convulsions, paroxysmal extreme pain disorder (PEPD), small fiber neuropathy. SCNC9A loss-of-functions mutations are associated with hereditary sensory autonomic neuropathy type 2D and congenital insensitivity to pain. Gain-of-function mutations, when inherited, are typically autosomal dominant, although increased severity in a compound heterozygote with a PEPD phenotype is reported [11]. Loss-of-function mutations typically are inherited in an autosomal recessive manner. Gain-of-function mutations alter the Nav1.7 channel function such that it is easier to activate, remains in the open state longer, and increases the response to a given stimulus [3, 10, 11, 12]. Despite this, drugs that act as Nav1.7 antagonists are not consistently helpful in treating erythromelalgia, although symptomatic relief in PEPD is commonly reported. SCN9A gain-of-function mutation-specific response to sodium channel antagonists is also confirmed in reports of in vitro studies [13]. Additionally, differential effects of given mutations on SCN9A-expressing cells sequestered in the DRGs versus those in sympathetic ganglia may also play a role in phenotype. This only serves to confirm the complexity of pain perception, processing, and modulation.

Our patient had relief with local anesthetic infusions intravenously and via regional techniques using axillary and lumbar epidural catheters. These did not represent long-term solutions. The team was aware of a report by Robbins et al. [14] using 5-10% compounded capsaicin cream to treat primarily chronic intractable foot pain of various etiologies, including complex regional pain syndrome type I, diabetic neuropathy, and ischemic neuropathy. After difficulties getting the 8% capsaicin patch to fully conform to the interspaces of the fingers and toes, it was decided to have our compounding pharmacy prepare a 10% capsaicin cream [15].

Capsaicin is a nonpolar phenolic compound found in chili peppers that is well absorbed through cell membranes when used topically [16]. It selectively interacts with C-polymodal nociceptors by binding to the transient receptor potential cation channel subfamily V member 1 (TRPV1) receptor causing the influx of cations, such as calcium, into dorsal root ganglion neurons. Neurons expressing TRPV1 receptors also contain proinflammatory neuropeptides, such as substance P and calcitonin gene-related peptide (CRGP), which are released upon stimulation and may play a role in both neurogenic inflammation and visceral pain in addition to nociception of elevated temperature and acidity [16, 17].

Despite its ability to cause pain, recurrent or concentrated doses of capsaicin can lead to analgesia. The exact mechanisms of its therapeutic effects are not fully known, but there is evidence to suggest that the TRPV1 receptors undergo a process of desensitization [16, 17]. Part of the process includes the depletion of neuropeptides [16] but can also be due to the phosphorylation of the TRPV1 receptor [18], causing a decreased responsiveness to subsequent stimuli. Furthermore, prolonged analgesic states could be explained by the ability of capsaicin to cause degeneration of sensory nerve fibers through apoptosis [16].

Our patient received five total treatments of 10% compounded capsaicin cream on her hands and feet. These treatments were performed under general anesthesia due to her age and anxiety at the time as well as the pain associated with the application. Each time, regional analgesia in the immediate post-application period was also provided via neural blockade with local anesthetic and clonidine. Her main complaint following application was a profound sense of pruritus that lasted for several days but was managed with diphenhydramine. The duration of symptom relief between the initial dose and the first reapplication was six months. This was followed by intervals of approximately 13 months, 16 months, 11 months, and now approaching eight years.

It is not unprecedented that multiple applications of topical capsaicin can lead to prolonged analgesia. Posttraumatic and postsurgical neuropathic pain of the chest wall was treated with six applications of the capsaicin 8% topical patch at 12-week intervals, ultimately leading to more than 18 months of sustained reduction of allodynia [19]. However, it is more likely that the eight-year duration of dramatic symptomatic improvement in our patient reflects the variable natural course of erythromelalgia. In an outcomes survey of patients diagnosed with erythromelalgia at the Mayo Clinic between 1970 and 1994, 39 (41.5%) out of 94 patients reported that their erythromelalgia symptoms had decreased or resolved entirely [20]. Currently, our patient’s symptoms are markedly reduced. She experiences momentary “shocks” daily and mini-flares lasting several hours during severe stress or illness. She is not on any medications for her erythromelalgia symptoms, is functioning exceptionally well as a college student, and only occasionally has issues with grip or hand strength.

Our patient’s overall neurologic phenotype is also interesting. Her initial electrodiagnostic studies were unrevealing but subtle abnormalities may have been masked by the cool temperature of her extremities. She experienced significant and persistent distal (hand) muscular atrophy. This may be at least partially explained by prolonged disuse during the initial phase of her symptoms, but her subsequent nerve conduction studies are for more pervasive peripheral nerve involvement. Still, the stability of her exam over nearly a decade afterward argues against a relentlessly progressive distal sensory-motor neuropathy. An initial distal severe neuropathic injury specific to the underlying disorder with incomplete recovery cannot be excluded, especially given the phenotypic spectrum of SCN9A-related disorders.

A recent report discusses two sisters with homozygous NMNAT2 mutations, distal motor neuropathy, and erythromelalgia [4]. Thus, not only is there allelic heterogeneity in SCN9A-related neuropathic pain disorders, but there is also genetic heterogeneity in erythromelalgia-associated phenotypes. Delineation of the molecular underpinnings in our case, as well as those with similar phenotypes, has implications for recurrence risk counseling, potentially for therapy and prognostication.

## Conclusions

In summary, erythromelalgia is an uncommon, variable, but well-defined disorder. The distinction between primary and secondary erythromelalgia is important as secondary erythromelalgia may reflect otherwise treatable underlying disorders.

Primary erythromelalgia, while often associated with mutations in SCN9A, is likely also genetically heterogeneous. Identification of a specific molecular cause in a given patient or family may have significant clinical implications. A diagnosis of primary erythromelalgia does not exclude possible more pervasive peripheral nerve involvement as part of the phenotype. Currently, however, consensus does not exist regarding “best practice” beyond supportive measures such as avoiding known triggers (e.g., heat). Sodium channel antagonists should probably be offered early for chronic if not acute relief, but myriad other agents may be reasonable options if that fails. Our case reflects not only the refractory nature of erythromelalgia and its highly variable course, including remission. More importantly, we provide a discussion of an effective acute management protocol for severe cases using synergistic regional and topical anesthetic and analgesic agents.
